# LASSBio-1586, an *N*-acylhydrazone derivative, attenuates nociceptive behavior and the inflammatory response in mice

**DOI:** 10.1371/journal.pone.0199009

**Published:** 2018-07-30

**Authors:** Juliane Cabral Silva, Raimundo Gonçalves de Oliveira Júnior, Mariana Gama e Silva, Érica Martins de Lavor, Juliana Mikaelly Dias Soares, Sarah Raquel Gomes de Lima-Saraiva, Tâmara Coimbra Diniz, Rosemairy Luciane Mendes, Edilson Beserra de Alencar Filho, Eliezer Jesus de Lacerda Barreiro, Lídia Moreira Lima, Jackson Roberto Guedes da Silva Almeida

**Affiliations:** 1 Núcleo de Estudos e Pesquisas de Plantas Medicinais (NEPLAME), Universidade Federal do Vale do São Francisco, Petrolina, Brasil; 2 Colegiado de Farmácia, Universidade Federal do Vale do São Francisco, Petrolina, Brasil; 3 Instituto Nacional de Ciência e Tecnologia de Fármacos e Medicamentos (INCT-INOFAR), Universidade Federal do Rio de Janeiro, Laboratório de Avaliação e Síntese de Substâncias Bioativas (LASSBio), Rio de Janeiro, Brasil; National University Singapore Yong Loo Lin School of Medicine, SINGAPORE

## Abstract

Pain and inflammation are complex clinical conditions that are present in a wide variety of disorders. Most drugs used to treat pain and inflammation have potential side effects, which makes it necessary to search for new sources of bioactive molecules. In this paper, we describe the ability of LASSBio-1586, an *N*-acylhydrazone derivative, to attenuate nociceptive behavior and the inflammatory response in mice. Antinociceptive activity was evaluated through acetic acid-induced writhing and formalin-induced nociception tests. In these experimental models, LASSBio-1586 significantly (*p<*0.05) reduced nociceptive behavior. Several methods of acute and chronic inflammation induced by different chemical (carrageenan, histamine, croton oil, arachidonic acid) and physical (cotton pellet) agents were used to evaluate the anti-inflammatory effect of LASSBio-1586. LASSBio-1586 exhibited potent anti-inflammatory activity in all tests (*p*<0.05). Study of the mechanism of action demonstrated the possible involvement of the nitrergic, serotonergic and histamine signaling pathways. In addition, a molecular docking study was performed, indicating that LASSBio-1586 is able to block the COX-2 enzyme, reducing arachidonic acid metabolism and consequently decreasing the production of prostaglandins, which are important inflammatory mediators. In summary, LASSBio-1586 exhibited relevant antinociceptive and anti-inflammatory potential and acted on several targets, making it a candidate for a new multi-target oral anti-inflammatory drug.

## Introduction

Pain is one of the most serious societal problems; it limits productivity and reduces quality of life as well as has high social and economic impacts. Pain is a complex experience involving emotional and neurobiological factors that normally indicate the presence of tissue damage to the organism. The pain response is established by the participation of several neurogenic or inflammatory mediators. During potential injury, chemical agents (histamine, bradykinin, prostaglandins, serotonin and nitric oxide) can activate and/or sensitize nociceptive fibers, contributing to the induction of pain and inflammation. In this context, inflammation can be understood to be a fundamental defense reaction of the body against the invasion of pathogens, injury and other noxious stimuli. Redness, warmth, swelling and pain are the classic clinical features of inflammation [1–3].

Currently, the pharmacological treatments used for the management of pain and inflammation include non-steroidal anti-inflammatory drugs (NSAIDs), glucocorticoids, central analgesics (opioids) and adjuvants drugs (anticonvulsants, antidepressants). Despite the great diversity of available anti-inflammatory and analgesic drugs, their side effects—for instance gastrointestinal disorders and ineffectiveness for some conditions—require a continuous search for new bioactive molecules. Therefore, management of pain/inflammation continues to be a major challenge for modern medicine. Thus, many studies focus on the search for new compounds as therapeutic alternatives. Neurotransmitters, receptors, and pharmacological targets have attracted much interest from the scientific community [4–6].

To explore new agents for the treatment of painful and inflammatory conditions, *N*-acylhydrazone (NAH) derivatives were studied in various pain and inflammation models. NAH is considered to be a privileged structure, as it is present in many compounds with diverse pharmacological activities. LASSBio-1586 is an example of an NAH derivative that was developed through molecular modification strategies. In a previous investigation, LASSBio-1586 was shown to inhibit microtubule polymerization, demonstrating a broad *in vitro* and *in vivo* antiproliferative profile with satisfactory selectivity for cancer cells [7].

In this sense and following the principle of drug repurposing, we describe in this paper the antinociceptive and anti-inflammatory potential of LASSBio-1586 in various experimental models. In addition, a docking study was performed with some of the main targets of the inflammatory process.

## Materials and methods

The *N*-acylhydrazone derivative LASSBio-1586 was obtained as previously described [7]. Briefly, methyl 3,4,5-trimethoxybenzoate was treated with hydrazine hydrate in ethanol to give 3,4,5-trimethoxybenzohydrazide. This key intermediate was treated with benzaldehyde in ethanol (7 ml) containing one drop of 37% hydrochloric acid. The mixture was stirred at room temperature for 1 hour and was then poured into ice. The precipitate was filtered and dried to give LASSBio-1586 in good yield. This compound was characterized, and its purity was determined using the methodologies described by Amaral and coworkers [7].

### Animals and ethics statement

All experiments were conducted using 8-week-old male Swiss mice (*Mus musculus*) (30–35 g). The total number of animals involved in this study was 300 animals, and each individual was involved in a single painfull procedure (not reused). The animals were kept in groups of six animals (n = 6) in polypropylene cages at a temperature of 22 ± 1°C and relative humidity of 60–80% with a light/dark cycle of 12:12 h (start 06:00 and end 18:00) and free access to food (Purina Labina^®^) and water. This study was performed in accordance with the Conselho Nacional para o Controle de Experimentação Animal (CONCEA, Brazil) and complied with the recommendations of the International Association for the Study of Pain [8]. The protocol was approved by the Comitê de Ética no Uso de Animais of the Universidade Federal do Vale do São Francisco (CEUA-UNIVASF, Brazil) with authorization number 0013/021014. All efforts were made to minimize animal suffering. At the end of the experiments, the animals were anesthetized with 60 mg/kg ketamine plus 7.5 mg/kg xylazine intraperitoneally and euthanized by cervical dislocation. For administration of all substances intraperitoneally syringes of 1 ml with needle of 13 x 0.45 mm were used. For the oral route a gavage needle was used.

### Acetic acid-induced writhing test

The writhing test was chosen as a classic model to assess the analgesic or anti-inflammatory properties of new agents [9]. This test was performed using the method described by Collier and collaborators [10] with modifications. Mice intraperitoneally (i.p.) received 10 ml/kg of a 0.9% acetic acid solution [11]. The amount of abdominal writhing was recorded for 10 min, beginning 5 min after administration of the acetic acid solution [12]. Writhing reflexes were defined as contractions of the abdominal muscles and pelvic rotation, followed by hind limb extension. Animals were divided into six groups of six animals each (n = 36) and were treated orally (p.o.) with LASSBio-1586 (10, 20 and 40 mg/kg) and saline (negative control) 1 h before the nociceptive agent. Indomethacin (20 mg/kg, i.p.) and morphine (10 mg/kg, i.p.) were used as reference drugs and administered 30 min before the acetic acid solution.

### Formalin-induced nociception test

The formalin test was performed as described by Hunskaar and Hole [13]. Animals were divided into six groups of six animals each (n = 36) and were treated with saline (p.o.), LASSBio-1586 (10, 20 and 40 mg/kg, p.o), indomethacin (20 mg/kg, i.p.) and morphine (10 mg/kg, i.p.) were given 1 h prior to the formalin injection. A formalin solution (2.5% in 0.9% sterile saline; 20 μl/animal) was injected into the right hind paw of mice [14]. Immediately after the formalin injection, animals were placed back in the chambers with a mirror and were observed for 30 min. The amount of time (in seconds) spent licking and biting the injected paw was measured as an indicator of pain. The formalin injection produced a biphasic nociceptive response: (I) an acute phase, 5 min after formalin injection, followed by a quiescent period of approximately 10 min and (II) a longer lasting tonic phase 15 to 30 min after this period [15].

To verify the possible involvement of the nitrergic and serotonergic systems in the pharmacological effect of LASSBio-1586, animals were divided into four groups of six animals each (n = 24) and were pretreated with the respective antagonists N(G)-Nitro-*L*-arginine methyl ester (*L*-NAME, 10 mg/kg, i.p) and ondansetron (0.5 mg/kg, i.p) 30 min before treatment with LASSBio-1586 (40 mg/kg, p.o) [16].

### Leukocyte migration to the peritoneal cavity induced by carrageenan

Leukocyte migration was induced with 250 μl of 1% carrageenan (i.p.) into the peritoneal cavity of mice 1 h after administration of saline (p.o.) and LASSBio-1586 (10, 20 and 40 mg/kg, p.o) and 0.5 h after injection of dexamethasone (2 mg/kg, i.p.). Animals (n = 30) were euthanized, as described above, 4 h later, and the peritoneal cavity was washed with 3 ml of a saline solution containing 1 mM EDTA [17]. The collected fluid was centrifuged (3000 rpm for 6 min) at room temperature. Subsequently, 10 μl of this suspension were dissolved in 200 μl of Turk solution, and a total cell count was performed using a Neubauer chamber. The results are expressed as the number of leukocytes/ml [18].

### Carrageenan-induced hind paw edema

Mice were divided into six groups of six animals each (n = 36) and were pretreated with LASSBio-1586 (10, 20 and 40 mg/kg, p.o), saline (p.o.) or indomethacin (20 mg/kg, i.p.) 1 h before subcutaneous injection of carrageenan (2.0% ƛ-carrageenan) or saline (0.9%) into the right hind paw of animals at a volume of 20 μl/animal [19,20]. The mice pedal volume up to the ankle joint was measured using a plethysmometer (PanLab LE 7500, Spain) 0, 1, 2, 3, 4 and 5 h after administration of carrageenan as described previously [21]. Inhibition of the paw edema was calculated by edema = (foot volume at measurement time—foot volume at time zero)/foot volume at time zero.

### Histamine-induced hind paw edema

To evaluate the involvement of histaminic receptors, mice were pretreated with LASSBio-1586 (40 mg/kg, p.o) or saline (p.o.) 1 h before the subcutaneous injection of histamine (100 μg/paw) or saline (0.9%) into their right hind paw at a volume of 20 μl/animal [22]. The volume was measured 0, 30, 60, 90 and 120 min after injection of histamine or saline [23].

### Croton oil-induced ear edema

In this model, inflammation was induced using 20 μl of 5% croton oil (v/v) in acetone on the inner and outer surfaces of the right ears, while the left ear received 20 μl of acetone. Animals (n = 30) were euthanized, as described above, six hours later, and a six-mm diameter plug was removed from both the treated and untreated ears with a punch. The edematous response was measured as the weight difference between the two plugs. Animals were pretreated with LASSBio-1586 (10, 20 and 40 mg/kg, p.o), saline (p.o.) or indomethacin (20 mg/kg, i.p.) 1 h before application of the irritant agent [24,25].

### Arachidonic acid-induced ear edema

One hour after treatment with LASSBio-1586 (10, 20 and 40 mg/kg, p.o), saline (p.o.) or indomethacin (20 mg/kg, i.p.) edema was induced by applying 20 μl of arachidonic acid to the inner and outer surface of the right ear, while the left ear received 20 μl of acetone and was used as a control. Thirty minutes after application of arachidonic acid, mice (n = 30) were euthanized as described above, and a six-mm diameter plug was removed from both the treated and untreated ears with a punch and weighed [26,27].

### Leukocyte migration into subcutaneous air pouches induced by carrageenan

Mice (n = 30) were pretreated with LASSBio-1586 (10, 20 and 40 mg/kg, p.o), saline (p.o.) or dexamethasone (2 mg/kg, i.p.) 1 h before subcutaneous injection of carrageenan (1.0% ƛ-carrageenan) into the air pouch. The air pouch was formed by injecting 10 ml of sterile air subcutaneously into the dorsal thoracic region. On day 6, carrageenan (0.25 ml) was injected into the pouch [28,29]. Six hours after this injection, the pouch was washed with 3 ml of PBS, and the collected fluid was centrifuged (3000 rpm for 6 min). Total cells were counted using a Neubauer chamber [30].

### Cotton pellet-induced granuloma

A granuloma was induced by sterile cotton pellets (10 ± 0.5 mg) implanted subcutaneously on the backs of mice 30 min after administration of saline (p.o.), LASSBio-1586 (10, 20 and 40 mg/kg, p.o.) and indomethacin (20 mg/kg, p.o.). Mice were divided into five groups of six animals each (n = 30). These animals were treated orally for six consecutive days. On day 7, the animals were anaesthetized and cotton pellets were removed surgically; any extraneous tissues were removed. The removed pellets were dried overnight at 60°C and weighed [31–34].

### Physico-chemical properties and ADMET profile

The physico-chemical properties and ADMET profile of LASSBio-1586 was predicted *in silico* in comparison with the anti-inflammatory drugs indomethacin and meloxicam, using the ACD/Percepta Program.

### Molecular docking analysis

The X-ray crystallographic structure of murine COX-2 enzyme, complexed with meloxicam (MXM), was obtained from the RCSB Protein Data Bank (PDB ID: 4M11) [35, 36]. This structure was chosen because of its structural similarity between LASSBio-1586 and the well-known NSAID meloxicam, which is most selective for the COX-2 isoform of cyclooxygenase. An analysis was performed using Autodock tools with the (ADT) v1.5.4 and Autodock v4.2 programs [37] (Autodock, Autogrid, Autotors, Copyright-1991–2000) produced by the Scripps Research Institute. The structure of LASSBio-1586 was initially constructed using ACD/ChemSketch 12.01 software [38]. Using the GaussView 6.0 and Gaussian 09 packages [39, 40], the 3D structure was adjusted similarly to the previously obtained crystal structure [7], followed by geometric optimization using the semi-empirical PM3 method. The structure of MXM was obtained directly from the original pdb complex. The preparation of docking simulations with LASSBio-1586 and MXM followed the same procedure. Gasteiger charges and polar hydrogens were assigned to protein and ligands. Nonpolar hydrogens were merged. Two water molecules present in the binding site [35] were kept and edited using the UCSF Chimera package [41]. The ligands were considered to be flexible upon analysis, and the rotatable bonds were chosen automatically by the program. To calculate the affinity maps used by Autodock, a grid point box with dimensions of 40 Å x 40 Å x 40 Å was initially chosen, with spacing of 0.375 Å between the points and centered on the ligand molecule (native MXM). A conformational search was performed with the Lamarckian Genetic Algorithm (LGA) [42]. The initial population was 150, with a maximum number of generations of 27,000. The maximum number of energy evaluations was 2,500,000 (long). The mutation and crossover rates were chosen as 0.02 and 0.8, respectively. At the end of the calculations, several conformations were placed into different clusters of similarity considering the binding energy and RMSD (Root Mean Square Deviation). The lowest-energy conformation of the more populated cluster was considered to be the most reliable solution. Based on the procedures described above, “redocking” was conducted considering the native MXM. The objective of this step was to determinate the accuracy of the docking procedure in this system, evaluating the RMSD (Root Mean Square Deviation) between the native and post-redocking conformation of MXM. The same step was conducted for LASSBio-1586 to view its possible interaction modes and binding energies.

### Statistical analysis

The results are presented as the mean ± standard error of the mean (SEM), and statistical analysis was performed using one-way analysis of variance (ANOVA) followed by Tukey’s test. Values of *p*<0.05 were considered statistically significant. All analyses were performed using GraphPad Prism^®^ 6.0 (Graph Pad Prism Software, Inc., San Diego, CA, USA).

## Results and discussion

**Fig 1:**
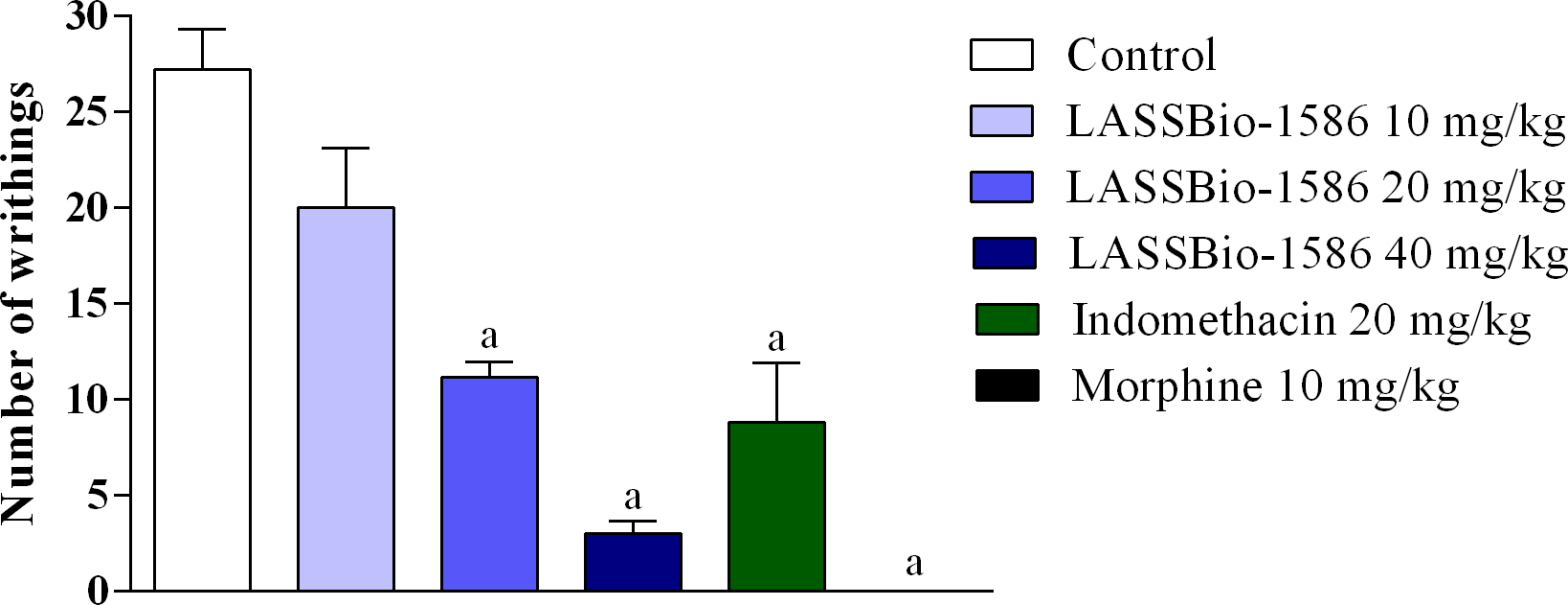
Effect of LASSBio-1586 (10, 20 and 40 mg/kg, p.o.), morphine (10 mg/kg, i.p.) and indomethacin (20 mg/kg, i.p.) in the acetic-acid-writhing-induced nociception test in mice (n = 6, per group). Values are expressed as the mean ± SEM, where *a* indicates *p*<0.05, significantly different from the control group, according to ANOVA, followed by Tukey’s test.

Initially, the antinociceptive potential of LASSBio-1586 was evaluated using the acetic acid-induced nociception test. In this model, LASSBio-1586 reduced nociceptive behavior in a dose-dependent manner, decreasing the number of writhings by up to 88.97% at the highest dose tested (40 mg/kg), as shown in Fig 1. This preliminary test indicated that LASSBio-1586 could be a promising antinociceptive agent. However, the acetic acid-induced writhing test is quite unspecific. Intraperitoneal administration of the chemical agent induces activation of nociceptors and stimulates the release of a variety of painful and inflammatory mediators, including histamine, bradykinin, serotonin, glutamate, noradrenaline, substance P, nitric oxide and prostaglandins [10]. For this reason, it is not possible to specify the nociceptive pathways in which the molecule acts. From this perspective, the formalin-induced nociception test was performed.

**Fig 2:**
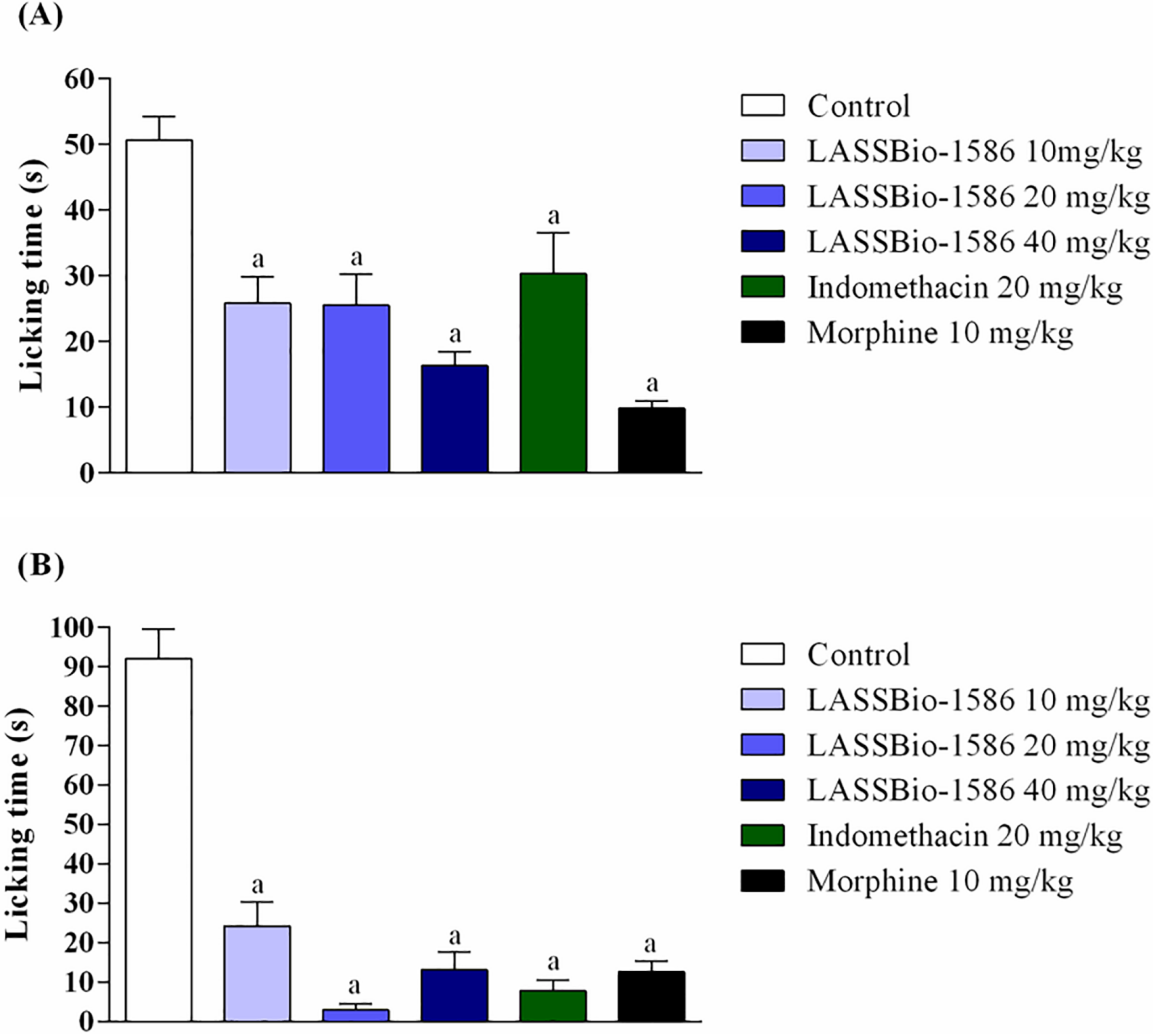
**Effect of LASSBio-1586 (10, 20 and 40 mg/kg, p.o.), morphine (10 mg/kg, i.p.) and indomethacin (20 mg/kg, i.p.) in the first (A) and second (B) phases of the formalin-induced nociception test in mice (n = 6, per group).** Values are expressed as the mean ± SEM, where *a* indicates *p*<0.05, significantly different from the control group, according to ANOVA, followed by Tukey’s test.

The formalin test is an experimental model that can verify the two distinct phases of a nociceptive episode. The first phase comprises a nociceptive response elicited by central-acting mediators, including those that activate serotonergic, muscarinic, vanilloid and glutamatergic receptors. In the second phase, there is a predominance of inflammatory mediators, mainly histamine, bradykinin and prostaglandins [13]. In this test, LASSBio-1586 reduced nociceptive behavior in both phases. LASSBio-1586 (10, 20 and 40 mg/kg) showed 49.01%, 49.67% and 67.77% of normal antinociceptive activity, respectively, in the first phase (Fig 2A). Similar to that observed for indomethacin, LASSBio-1586 promoted a better pharmacological effect in the second phase of the test, reaching 96.74% of antinociceptive activity at a dose of 20 mg/kg (Fig 2B).

**Fig 3:**
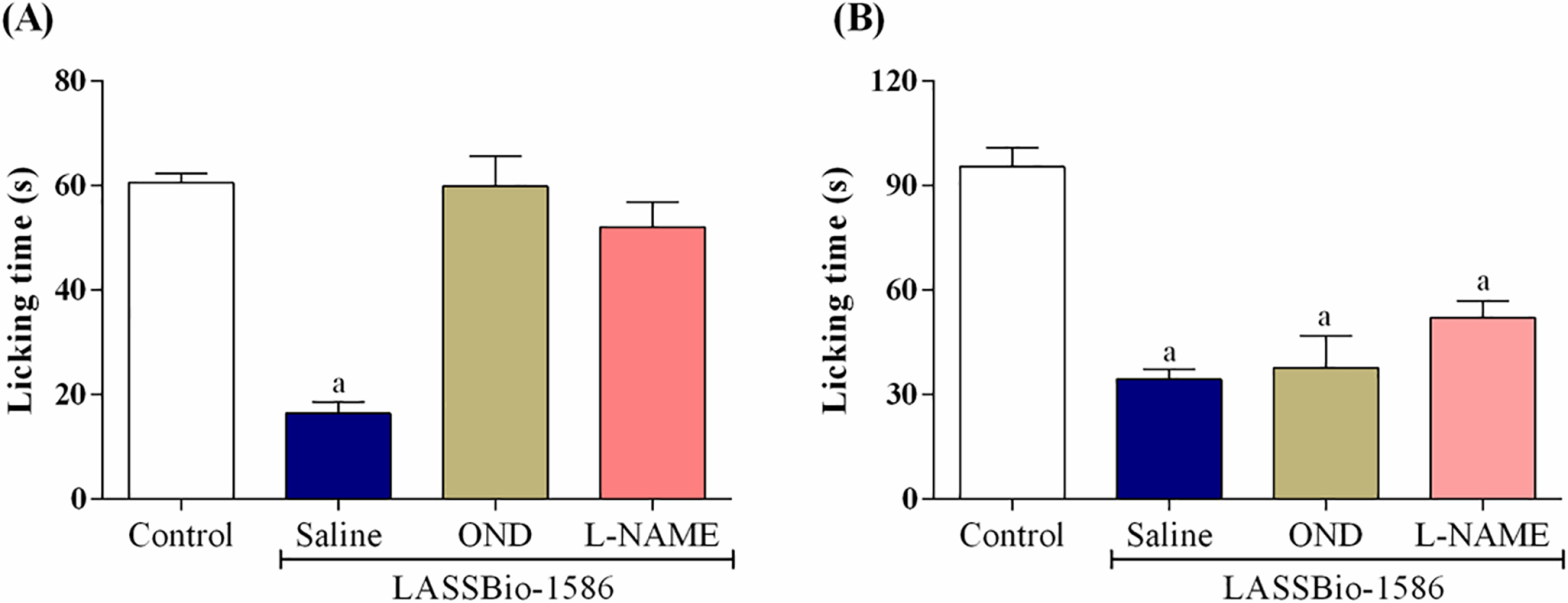
**Effect of LASSBio-1586 (40 mg/kg, p.o.), LASSBio-1586 (40 mg/kg, p.o.) + ondansetron (OND 0.5 mg/kg, i.p.) and LASSBio-1586 (40 mg/kg, p.o.) + L-NAME (10 mg/kg, i.p.) in the first (A) and second (B) phases of the formalin-induced nociception test in mice (n = 6, per group).** Values are expressed as the mean ± S.E.M., where *a* indicates *p*<0.05, significantly different from the control group, according to ANOVA, followed by Tukey’s test.

When animals were pretreated with ondansetron or *L*-NAME, the pharmacological effect of LASSBio-1586 (40 mg/kg, p.o.) was completely reversed in the first phase of the test (Fig 3), suggesting that its central antinociceptive response was involved at least in part in the serotoninergic and nitrergic systems. Serotonin [5-hydroxytryptamine (5-HT)] has long been considered to have an important role in the control of pain in the central nervous system, particularly through descending inhibition. Recently, many pharmacological investigations have focused on the role played by 5-HT in acute or chronic pain conditions, as well as the identification of the respective 5-HT receptors that are involved. In general, 5-HT_1A_ and 5-HT_7_ receptor agonists may be promising therapeutic agents for the treatment of pain states [43–45].

Recent pharmacological reports have shown that nitric oxide (NO) in the nociceptive response has a dual effect on the regulation of pain mechanisms and can act as a nociceptive or antinociceptive agent, depending on the cell conditions. Experimental data have demonstrated that NO inhibits nociception in the peripheral and central nervous systems. Normally, the analgesic action of NO depends on an intracellular signaling pathway that includes the formation of cyclic GMP, activation of PKG and consequent opening of K^+^ channels. The opening of these channels increases the K^+^ current, leading to hyperpolarization of nociceptive neurons [46,47]. From this point of view, LASSBio-1586 induces production of NO and consequently inhibits the nociceptive response.

**Fig 4:**
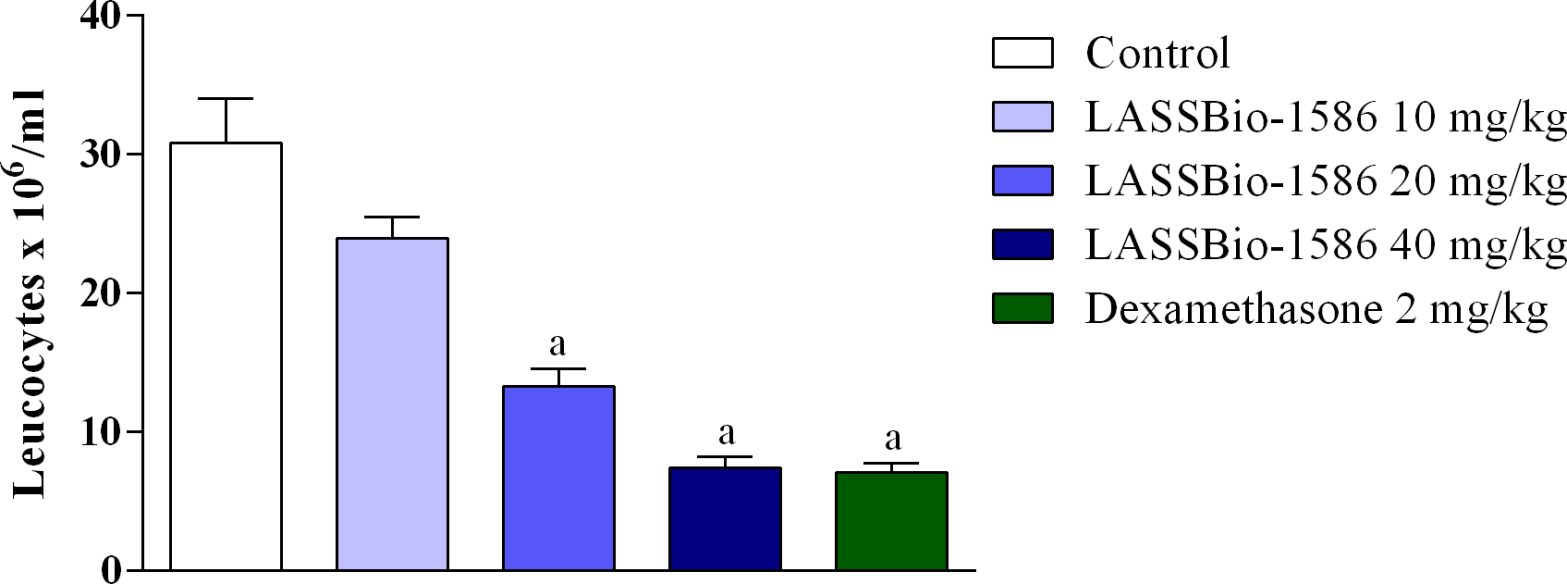
Effect of LASSBio-1586 (10, 20 and 40 mg/kg, p.o.) and dexamethasone (2 mg/kg, i.p.) on leukocyte migration into the peritoneal cavity induced by carrageenan in mice. Values are expressed as the mean ± S.E.M. (n = 6, per group), where *a* indicates *p*<0.05, significantly different from the control group, according to ANOVA, followed by Tukey’s test.

Although LASSBio-1586 reduced nociceptive behavior in both phases of the formalin test, the pharmacological response was more intense in the second phase, indicating that the antinociceptive effect of our NAH derivative was related to a reduction in inflammation. In this context, we conducted several tests to explore the anti-inflammatory potential of LASSBio-1586. At first, the anti-inflammatory effect of LASSBio-1586 was evaluated in models of acute inflammation, such as the leukocyte migration to the peritoneal cavity induced by carrageenan test. In this method, LASSBio-1586 reduced leukocyte migration in a dose-dependent manner (Fig 4). At the highest dose (40 mg/kg, 75.89%), the anti-inflammatory effect was equivalent to that observed for dexamethasone (2 mg/kg, 76.96%).

**Fig 5:**
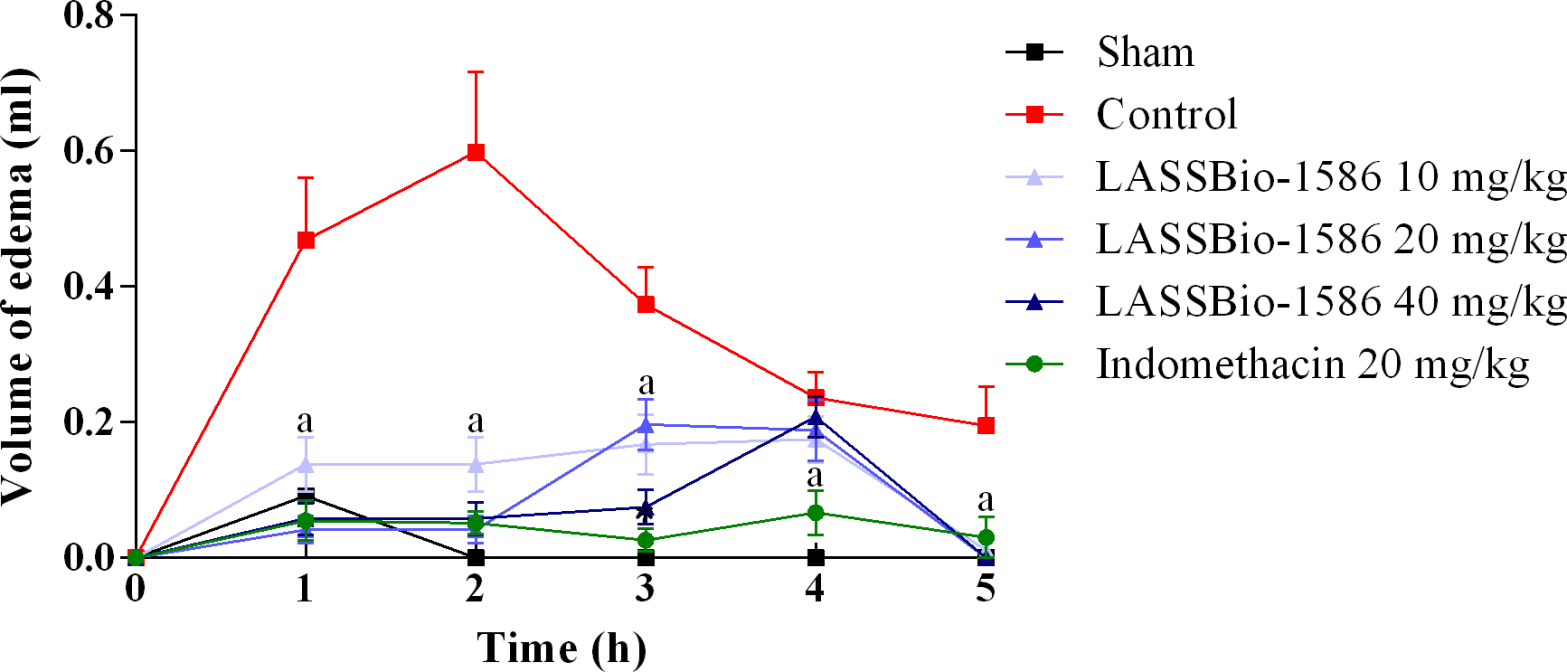
Effect of LASSBio-1586 (10, 20 and 40 mg/kg, p.o.) and indomethacin (20 mg/kg, i.p.) on paw edema induced by carrageenan in mice. The sham group was treated only with saline, whereas the control group received saline and carrageenan. Values are expressed as the mean ± S.E.M. (n = 6, per group), where *a* indicates *p*<0.05, significantly different from the control group, according to ANOVA, followed by Tukey’s test.

In the carrageenan-induced hind paw edema model, LASSBio-1586 significantly decreased (*p*<0.05) paw edema at all doses tested, especially at 1, 2 and 3 hours after treatment, suggesting strong anti-inflammatory activity, as shown in Fig 5. In the first few hours of the test, inflammatory mediators are released in response to carrageenan injection. Histamine is one of the first mediators produced since it is a fast-acting vasoactive amine that promotes vessel dilation from local circulation, which is essential for exudation and edema formation [19].

**Fig 6:**
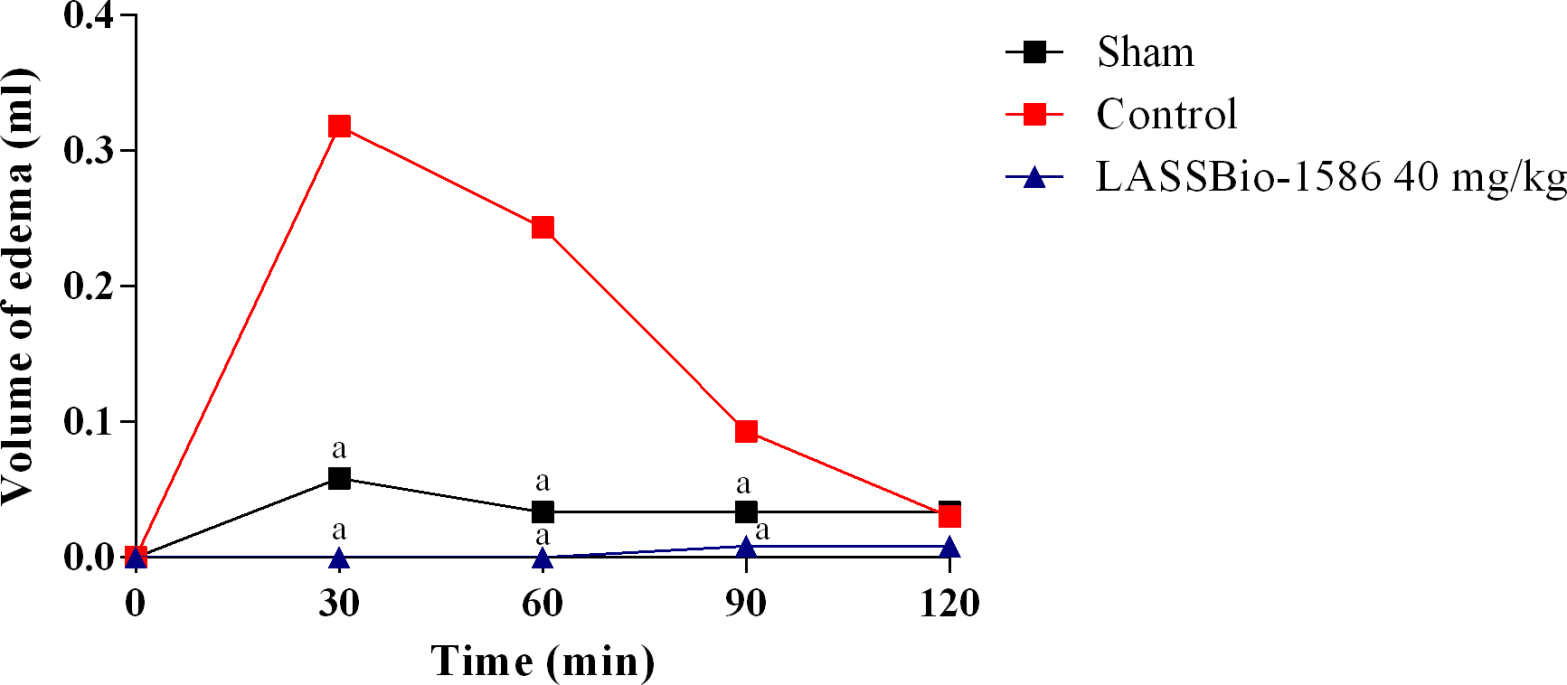
Effect of LASSBio-1586 (40 mg/kg, p.o.) on paw edema induced by histamine in mice. The sham group was treated only with saline, whereas the control group received saline and histamine. Values are expressed as the mean ± S.E.M. (n = 6, per group), where *a* indicates *p*<0.05, significantly different from the control group, according to ANOVA, followed by Tukey’s test.

In this sense, to assess the role of histaminic receptors in the anti-inflammatory effect of LASSBio-1586, a similar protocol was performed using histamine as a paw edema inducer. Fig 6 shows that LASSBio-1586 (40 mg/kg, p.o.) significantly reduced (*p*<0.05) histamine-induced paw edema at 30, 60 and 90 minutes, suggesting the involvement of histamine receptors in its anti-inflammatory effect.

**Fig 7:**
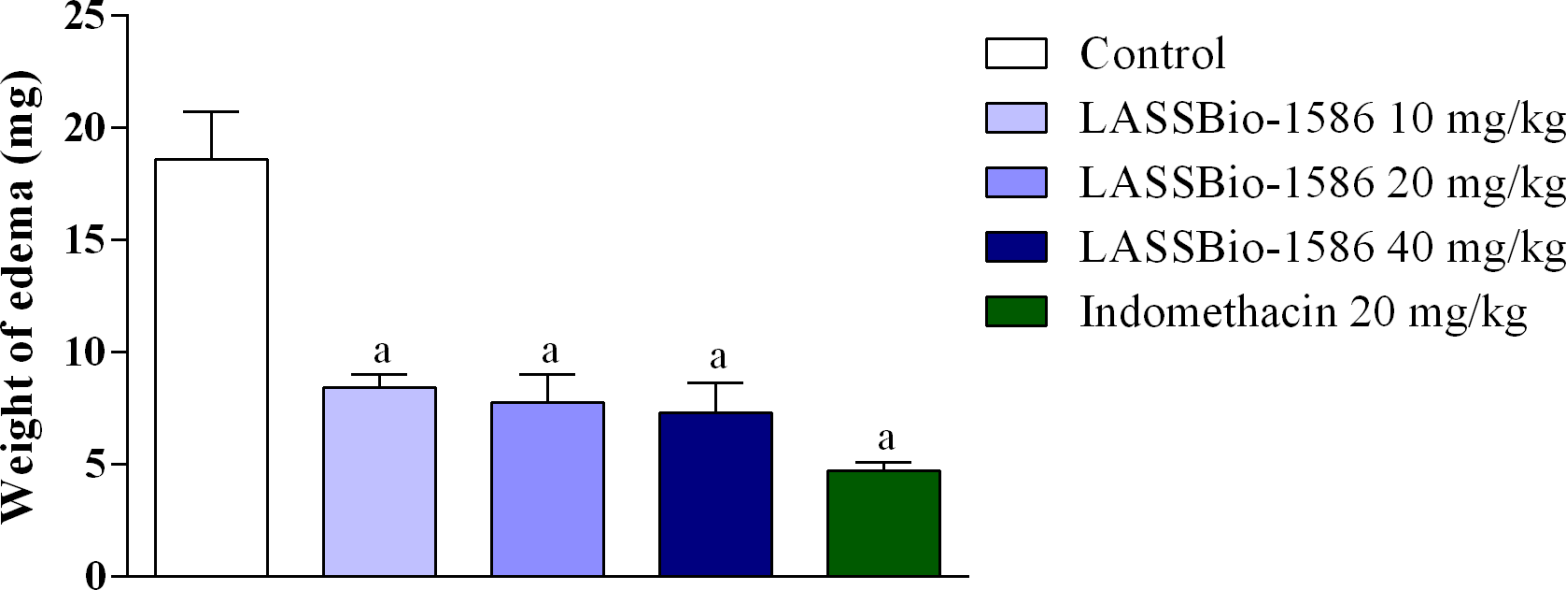
Effect of LASSBio-1586 (10, 20 and 40 mg/kg, p.o.) and indomethacin (20 mg/kg, i.p.) on croton oil-induced ear edema test in mice. Values are expressed as the mean ± S.E.M (n = 6, per group), where *a* indicates *p*<0.05, significantly different from the control group, according to ANOVA, followed by Tukey’s test.

The anti-inflammatory effect of LASSBio-1586 was also evaluated against the topical application of inflammatory agents. In the croton oil-induced ear edema test, animals treated with LASSBio-1586 presented a significant reduction in the weight of edema at all tested doses. A similar result was found for indomethacin-treated mice, as shown in Fig 7. These findings indicate the ability of LASSBio-1586 to attenuate the inflammatory process, induced by various chemical agents and routes of administration, making it a strong candidate for a new anti-inflammatory drug.

**Fig 8:**
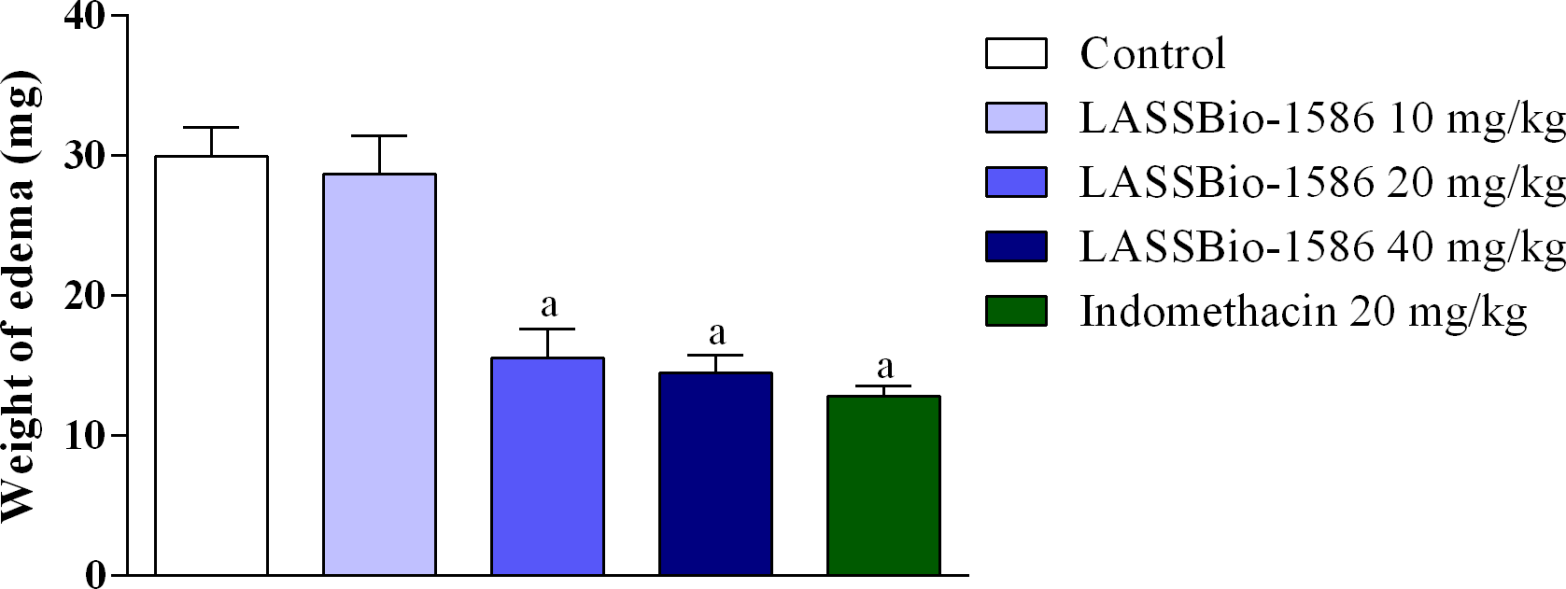
Effect of LASSBio-1586 (10, 20 and 40 mg/kg, p.o.) and indomethacin (20 mg/kg, i.p.) on the arachidonic acid-induced ear edema test in mice. Values are expressed as the mean ± S.E.M (n = 6, per group), where *a* indicates *p*<0.05, significantly different from the control group, according to ANOVA, followed by Tukey’s test.

Chemical-induced ear edema is an extremely versatile experimental model, and several agents can be used to investigate the involvement of various signaling pathways. Given the importance of prostaglandin production to maintain inflammation and promote the sensitization of nociceptive fibers [48,49], we conducted this experiment using arachidonic acid as an inducer of ear edema. In this test, LASSBio-1586 reduced ear edema at higher doses (20 and 40 mg/kg, p.o.), demonstrating approximately 47.93 and 51.53% of anti-inflammatory activity (Fig 8).

Arachidonic acid is a major product obtained after degradation of membrane phospholipids by the phospholipase A_2_ enzyme. Once produced, arachidonic acid serves as a substrate for cyclooxygenase enzymes, producing a diversity of prostaglandins (PGE_2_, PGI_2_, PGD_2_, PGF_2_) [49,50]. The significant reduction in ear edema promoted by LASSBio-1586 indicates its potential for inhibiting the degradation of arachidonic acid into inflammatory mediators, such as prostaglandins.

**Fig 9:**
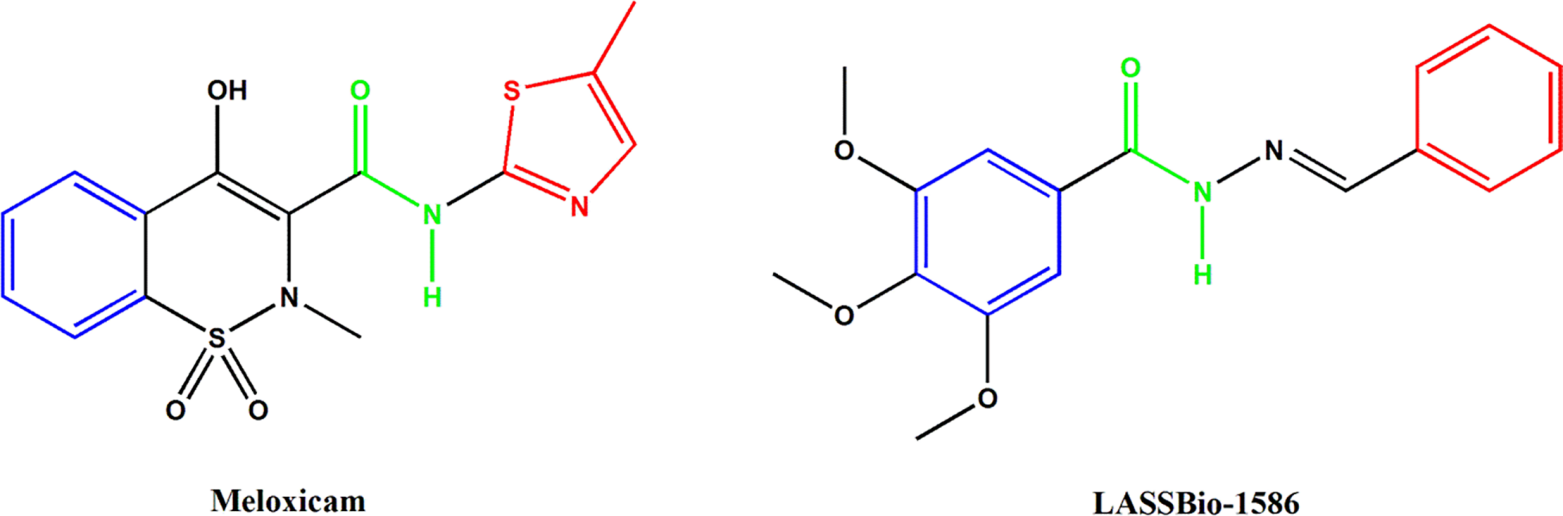
2D comparative representation of the NSAID meloxicam and LASSBio-1586.

To examine the hypothesis involving inhibition of prostaglandins production in greater detail, we performed a molecular docking study to investigate the interaction of LASSBio-1586 with the COX-2 cyclooxygenase isoform. From a molecular standpoint, it is interesting that LASSBio-1586 is structurally similar to the well-known nonsteroidal anti-inflammatory drug (NSAID) meloxicam, a drug that has high selectivity for COX-2. The similarity includes molecular length, presence of aromatic rings spaced by a chain with an amide-like bond in similar regions and tendency of the molecules to be planar due to conjugated double extension (Fig 9).

**Fig 10:**
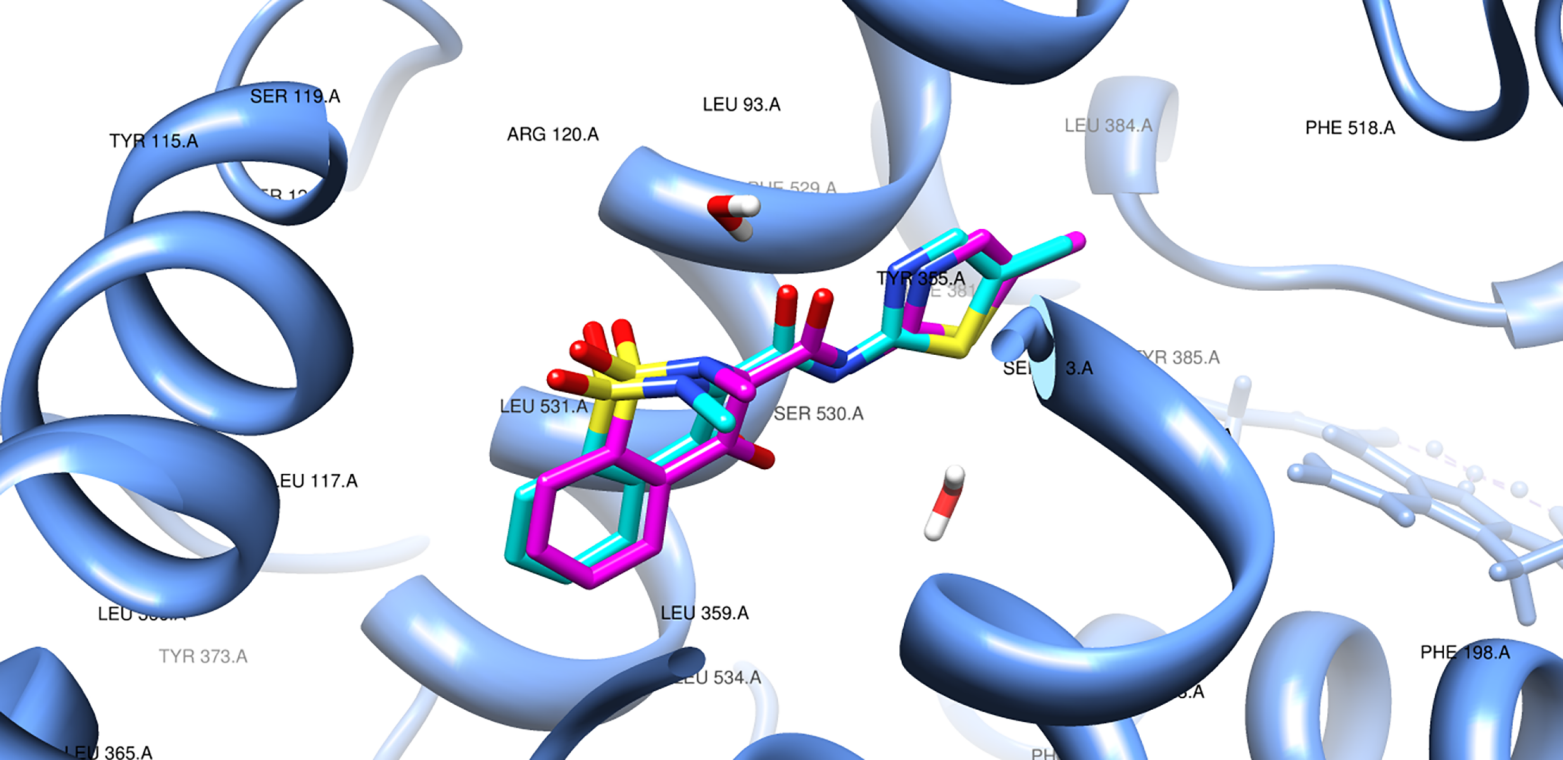
Native (magenta) and post-redocking (cyan) poses of MXM/murine COX-2 complex. RMSD = 0.61 Å. Binding Energy = −9.82 Kcal/mol.

In recent years, the crystallographic structure of murine COX-2 (*Mus musculus*) complexed with meloxicam was deposited in the online PDB database. The enzyme contains four monomers with identical residues that are complexed with four meloxicam molecules. The conformation of the “A” monomer was considered for docking procedures. The redocking of native MXM presented an RMSD = 0.61 Å, with Binding Energy = −9.82 Kcal/mol, for the best pose. This result validates the use of AutoDock for this complex, as the maximum value accepted in the literature to the RMSD is 2.0 Å [51]. Fig 10 shows that the redocking and native conformations are very similar.

**Fig 11:**
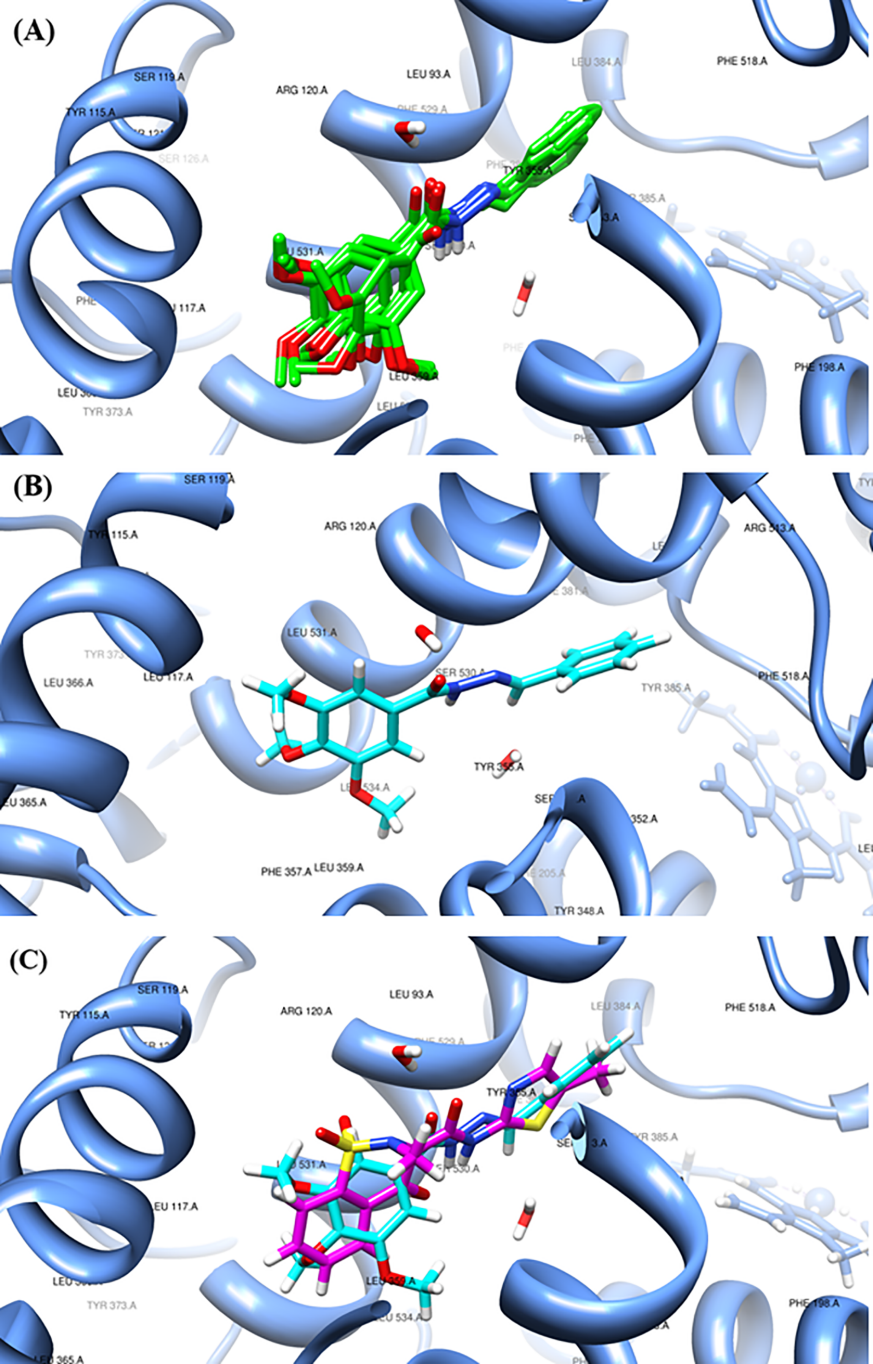
Results of the docking procedures for LASSBio-1586. **(A)** Conformations obtained from the most populous and stable cluster in the binding site of COX-2; **(B)** most stable docked conformation for LASSBio-1586 (Binding Energy = −7.84 Kcal/mol); **(C)** native conformation of meloxicam (magenta) and most stable conformation for LASSBio-1586 (cyan).

The best docking poses presented by LASSBio-1586 provided interesting insight into the structure and analgesic/anti-inflammatory activity relationships. Fig 10 show a sequence of images, and the five best poses for LASSBio-1586 are very similar (Fig 11A). The pose with the lowest binding energy (−7.84 Kcal/mol) is also shown (Fig 11B), as is a comparison performed by overlapping MXM and LASSBio-1586 after docking (Fig 11C).

Oxicams are a structurally unique class of NSAIDs. Studies of crystal complexes of meloxicam and murine COX-2 [35] showed that this class interacts in a novel mode compared to other NSAIDs (naproxen, diclofenac, among others), which occupy the entire channel that interacts with the enzyme, as mediated by two water molecules. In the structure proposed in Fig 11C, we observe that the hydrazone group of LASSBio-1586 is arranged in the same manner as the meloxicam amide group, establishing the same water-mediated interactions: the alpha-nitrogen of hydrazone in LASSBio-1586 occupies a similar position as that of the nitrogen of meloxicam and is able to interact with Ser-530; the carbonyl oxygen of hydrazone in LASSBio-1586 occupies a similar position as the amide oxygen in MXM, which binds Arg-120 and Tyr-355 via another ordered water molecule [35], reinforcing the idea that meloxicam and LASSBio-1586 have a similar pattern of recognition (Fig 11). However, it is also known that oxicam drugs contain a heteroatom at the 1’ position of the carboxamide five-membered ring (a nitrogen atom in meloxicam, Fig 9), which establishes water-mediated interactions with Tyr-385 and Ser-530 [35]. The absence of this interaction in LASSBio-1586 could explain its less effective binding energy compared to the redocking pose of MXM.

Binding of meloxicam is accompanied by an exclusive interaction between the phenyl ring and side chain of Leu-531 [35]. Other amino acids complete this hydrophobic pocket as Leu-534, Ile-345 and Val-344. This molecular volume of occupation is observed in the best poses of docked LASSBio-1586, considering the trimethoxy-substituted aromatic ring (Fig 11). In the same way, the original crystallographic article indicated the interaction between Phe-518 and the methyl group at the 4’ position of the meloxicam thiazole ring as being an important interaction with COX-2. Observing the proposed structure in Fig 11, we note that the unsubstituted phenyl ring of LASSBio-1586 occupies the same region as methyl thiazole, with an additional possible π-stacking interaction involving the aromatic ring of Phe-518. These crystallographic and theoretical data at the molecular level corroborate the hypothesis of the analgesic/anti-inflammatory effect of LASSBio-1586 in mice, at least in part via the COX-2 inhibition pathway.

**Fig 12:**
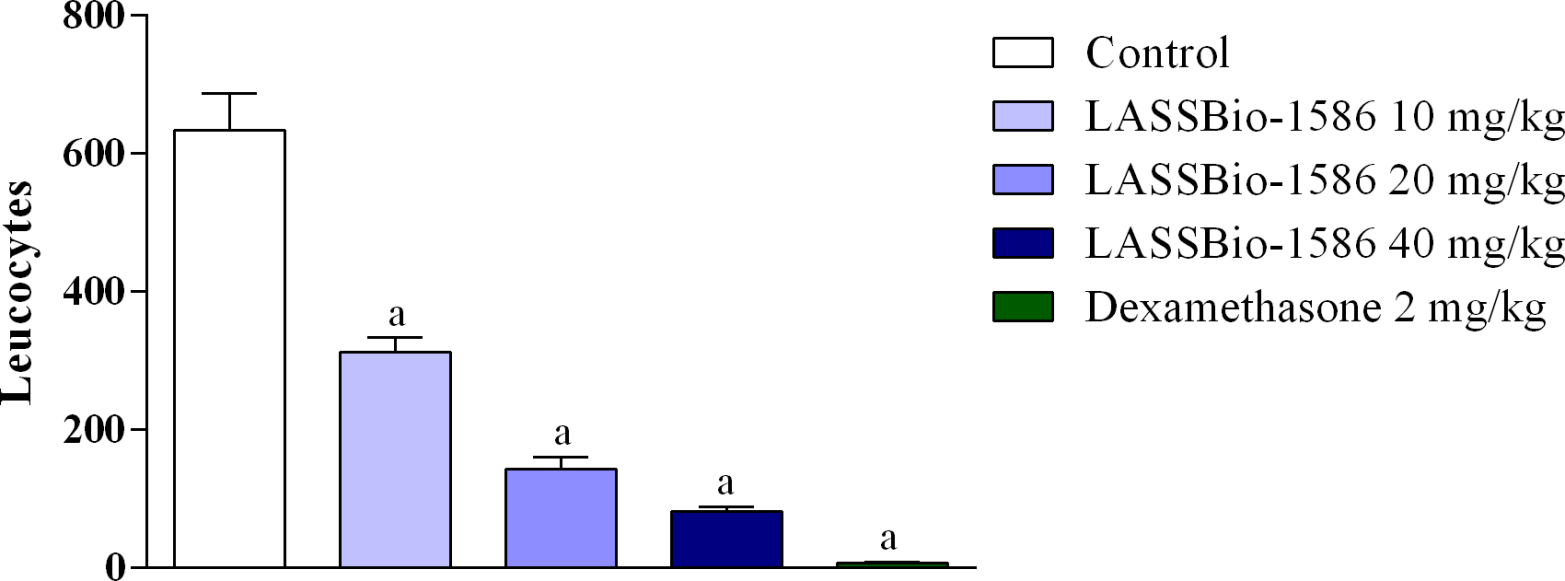
Effect of LASSBio-1586 (10, 20 and 40 mg/kg, p.o.) and dexamethasone (2 mg/kg, i.p.) on leukocyte migration into subcutaneous air pouches induced by carrageenan in mice. Values are expressed as the mean ± S.E.M (n = 6, per group), where *a* indicates *p*<0.05, significantly different from the control group, according to ANOVA, followed by Tukey’s test.

**Fig 13:**
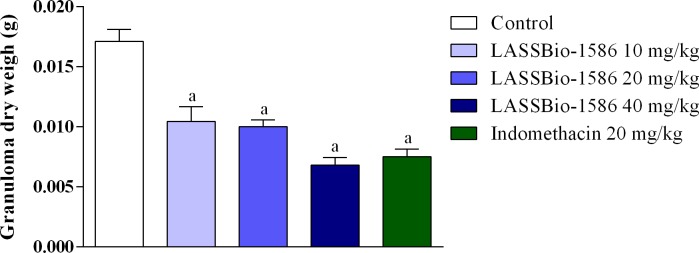
Effect of LASSBio-1586 (10, 20 and 40 mg/kg, p.o.) and indomethacin (20 mg/kg, p.o.) on cotton pellet-induced granuloma. Values are expressed as the mean ± S.E.M. (n = 6, per group), where *a* indicates *p*<0.05, significantly different from the control group, according to ANOVA, followed by Tukey’s test.

Finally, we evaluated the anti-inflammatory potential of LASSBio-1586 in chronic inflammation models. Fig 12 shows the pharmacological response of LASSBio-1586 on leukocyte migration into subcutaneous air pouches induced by carrageenan. In this test, our NAH derivative significantly reduced the number of leukocytes at all doses tested (10, 20 and 40 mg/kg, p.o.), presenting 50.63, 77.39 and 87.03% of the untreated anti-inflammatory effect, respectively. Similarly, LASSBio-1586 also decreased cotton pellet-induced granuloma, as demonstrated in Fig 13. These findings demonstrate the ability of LASSBio-1586 to combat the inflammatory response induced by different agents and at several stages, making it a strong candidate for a new oral anti-inflammatory drug.

Concerning the toxicity, preliminary studies have shown that LASSBio-1586 was capable of preventing microtubule polymerization and showed an extensive *in vitro* antiproliferative profile, as well as a better selectivity index, indicating improved selective cytotoxicity toward cancer cells unlike non-tumor cells [7]. This may suggest that the LASSBio-1586 has low toxicity to non-tumor cells, presenting a good safety profile in vitro. However, we agree that future toxicological evaluation is necessary to confirm the potential of LASSBio-1586 as a candidate drug for the treatment of inflammatory diseases and pain disorders.

Regarding to the physico-chemical properties and ADMET profile, as demonstrated in Table 1, no violation to Lipinski rule of five (Ro5) was found for LASSBio-1586, indicating that this compound has properties that would make it a likely orally active drug in humans [52].

**Table 1:** Comparative *in silico* physico-chemical properties and ADMET profile of LASSBio-1586 and the anti-inflammatory drugs indomethacin and meloxicam.

Predicted Properties*	Compounds		
	LASSBio-1586	Indomethacin	Meloxicam
MW (g/mol)	314.34	357.79	351.40
H-Donors	1	1	2
H-Acceptors	6	5	7
Rotatable Bonds	6	4	2
TPSA	69.15	68.53	136.22
LogP	2.44	4.02	2.38
Solubility	0.002 mg/ml	2.5 mg/ml	5.96 mg/ml
Caco-2	*P*_*e*_ = 231 ×10^−6^ cm/s	*P*_*e*_ = 129 ×10^−6^ cm/s	*P*_*e*_ = 233 ×10^−6^ cm/s
HIA	100%	100%	100%
F (oral)	80%	99%	96%
PPB	93%	99%	99%
CNS score	-2.72	-4.32	-5.24
HLM	0.49	0.26	0.32
hERG	0.52	0.23	0.41
AMES	0.42	0.27	0.21

*Determined *in silico* using the ACD/Percepta Program.

MW = molecular weight; H-Donors = hydrogen bond-donors; H-Acceptors = hydrogen bond-acceptors; TPSA = topological polar surface area; LogP = the logarithm of the drug partition coefficient between n-octanol and water; Caco-2 = human epithelial cell line Caco-2; HIA = human intestinal absorption; F = Bioavailability; CNS = central nervous system; HLM = human liver microsomes; hERG = the human Ether-à-go-go-Related Gene; AMES = Ames test = *Salmonella typhimurium* reverse mutation assay.

However, LASSBio-1586 was predicted to be an insoluble drug once compared to indomethacin and meloxicam. Regarding their comparative ADMET profile, they were predicted to be highly absorbed (HIA = 100%), highly permeable (Pe > 7 ×10^−6^ cm/s) and extensively bound to plasma protein (PPB > 90%). All compounds have showed great oral bioavailability *in silico* (F = 80–99%). The main differences amongst LASSBio-1586, indomethacin and meloxicam rely on their metabolic stability in human liver microsomes (HLM) and their ability to penetrate in CNS. As depicted in Table 1, indomethacin and meloxicam were predicted as stable in HLM (scores of 0.26 and 0.32, respectively), while an undefined result was found for LASSBio-1586 (Score = 0.49). Both anti-inflammatory drugs have been predicted as non-penetrant to CNS (scores of -4.32 and -5.24, respectively) whereas LASSBio-1586 was defined as able to penetrate CNS. To have some clues about the toxicity of LASSBio-1586, the ability of this compound to inhibit hERG (the human Ether-à-go-go-Related Gene), and its mutagenic profile (i.e. probability of a positive Ames test) were predicted. The results were converted into classification scores, and an undefined hERG and mutagenic activities (score > 0.33 and ≤ 0.67) were predicted for LASSBio-1586. Taken together, those *in silico* results suggest an adequate pharmacokinetic profile for LASSBio-1586, meanwhile the *in silico* approach could not predicted its toxicological profile.

## Conclusion

Based on the results, LASSBio-1586 showed significant antinociceptive and anti-inflammatory activities in all of the experimental models. Its mechanism of action appears to involve the serotonergic, nitrergic and histaminic signaling pathways, in addition to inhibition of COX-2, as demonstrated by the molecular docking study. In summary, LASSBio-1586 has emerged as a strong candidate for a multi-target anti-inflammatory drug.

## Supporting information

S1 Dataset Raw data. (RAR)

## References

[1] Pace MC, Mazzariello L, Passavanti MB, Sansone P, Barbarisi M, Aurilio C. Neurobiology of pain. J Cell Physiol. 2006;209: 8–12. 10.1002/jcp.20693 16741973

[2] Brito RG, dos Santos PL, Quintans JS, de Lucca Júnior W, Araújo AA, Saravanan S, et al Citronellol, a natural acyclic monoterpene, attenuates mechanical hyperalgesia response in mice: Evidence of the spinal cord lamina I inhibition. Chem Biol Interact. 2015;239: 111–117. 10.1016/j.cbi.2015.06.039 26141506

[3] Almeida AAC, Silva RO, Nicolau LAD, Brito TV, Sousa DP, Barbosa ALR, et al Physio-pharmacological Investigations about the Anti-inflammatory and Antinociceptive Efficacy of (+)-Limonene Epoxide. Inflammation. 2017;40: 511–522. 10.1007/s10753-016-0496-y 28091830

[4] Wang Y, Chen P, Tang C, Wang Y, Li Y, Zhang H. Antinociceptive and anti-inflammatory activities of extract and two isolated flavonoids of *Carthamus tinctorius* L. J Ethnopharmacol. 2014;151: 944–950. 10.1016/j.jep.2013.12.003 24333963

[5] Souza GV, Simas AS, Bastos-Pereira AL, Frois GRA, Ribas JLC, Verdan MH, et al Antinociceptive Activity of the Ethanolic Extract, Fractions, and Aggregatin D Isolated from *Sinningia aggregata* Tubers. PLoS One. 2015;10(2): e0117501 10.1371/journal.pone.0117501 25719394 PMC4342217

[6] Xu J, Zhao Q, Wei L, Yang Y, Xu R, Yu N, et al Phytochemical Composition and Antinociceptive Activity of *Bauhinia glauca* subsp. *hupehana* in Rats. PLoS One. 2015;10(2): e0117801 10.1371/journal.pone.0117801 25658740 PMC4320050

[7] do Amaral DN, Cavalcanti BC, Bezerra DP, Ferreira PM, Castro RP, Sabino JR, et al Docking, synthesis and antiproliferative activity of *N*-acylhydrazone derivatives designed as combretastatin A4 analogues. PLoS One. 2014;9: e85380 10.1371/journal.pone.0085380 24614859 PMC3948622

[8] Zimmermann M. Ethical guidelines for investigations of experimental pain in conscious animals. Pain. 1983;16: 109–110. 6877845 10.1016/0304-3959(83)90201-4

[9] Mohamad AS, Akhtar MN, Zakaria ZA, Perimal EK, Khalid S, Mohd PA, et al Antinociceptive activity of a synthetic chalcone, flavokawin B on chemical and thermal models of nociception in mice. Eur J Pharmacol. 2010;647: 103–109. 10.1016/j.ejphar.2010.08.030 20826146

[10] Collier HO, Dinneen LC, Johnson CA, Schneider C. The abdominal constriction response and its suppression by analgesic drugs in the mouse. Brit J Pharmacol Chemother. 1968;32: 295–310. 4230818 10.1111/j.1476-5381.1968.tb00973.x PMC1570212

[11] Silva JC, Araújo CS, Lima-Saraiva SRG, Oliveira-Junior RG, Diniz TC, Wanderley CW, et al Antinociceptive and anti-inflammatory activities of the ethanolic extract of *Annona vepretorum* Mart. (Annonaceae) in rodents. BMC Complement Altern Med. 2015;15: 197 10.1186/s12906-015-0716-2 26104689 PMC4478715

[12] Aoki M, Tsuji M, Takeda H, Harada Y, Nohara J, Matsumiya T, et al Antidepressants enhance the antinociceptive effects of carbamazepine in the acetic acid-induced writhing test in mice. Eur. J. Pharmacol. 2006;550: 78–83. 10.1016/j.ejphar.2006.08.049 17027750

[13] Hunskaar S, Hole K. The formalin test in mice: dissociation between inflammatory and non-inflammatory pain. Pain. 1987;30: 103–114. 3614974 10.1016/0304-3959(87)90088-1

[14] Santos DA, Fukuib MJ, Nanayakkarac NPD, Khan SI, Sousa JP, Bastos JK, et al Anti-inflammatory and antinociceptive effects of *Baccharis dracunculifolia* DC (Asteraceae) in different experimental models. J Ethnopharmacol. 2010;127: 543–550. 10.1016/j.jep.2009.09.061 19808087

[15] Holanda ADV, Asth L, Santos AR, Guerrini R, Soares-Rachetti VP, Calo' G, et al Central adenosine A1 and A2A receptors mediate the antinociceptive effects of neuropeptide S in the mouse formalin test. Life Sciences. 2015;120: 8–12. 10.1016/j.lfs.2014.10.021 25447449

[16] Leite LHI, Leite GO, Coutinho TS, Sousa SDG, Sampaio RS, Costa JGM, et al Topical Antinociceptive Effect of *Vanillosmopsis arborea* Baker on Acute Corneal Pain in Mice. J Evid Based Complementary Altern Med. 2014;2014: 1–6. 10.1155/2014/708636 PMC3934451 24660017

[17] Pereira JG, Mesquita JX, Aragão KS, Franco AX, Souza MHLP, Brito TV, et al Polysaccharides isolated from *Digenea simplex* inhibit inflammatory and nociceptive responses. Carbohyd Polym. 2014;108: 17–25. 10.1016/j.carbpol.2014.01.105 24751242

[18] Melo MS, Guimarães AG, Santana MF, Siqueira RS, Lima ACB, Dias AS, et al Anti-inflammatory and redox-protective activities of citronellal. Biol Res. 2011;44: 363–368. doi: /S0716-97602011000400008 22446600

[19] Winter CA, Risley EA, Nuss GW. Carrageenan-induced oedema in hind paw of rat as an assay for anti-inflammatory drugs. Proc Soc BioI Med. 1962;111: 544–547. 10.3181/00379727-111-27849 14001233

[20] Yamazaki Y, Yasuda K, Matsuyama T, Ishihara T, Higa R, Sawairi T, et al A *Penicillium* sp. F33 metabolite and its synthetic derivatives inhibit acetyl-CoA:1-O-alkyl-sn-glycero-3-phosphocholine acetyltransferase (a key enzyme in platelet-activating factor biosynthesis) and carrageenan-induced paw edema in mice. Biochem Pharmacol. 2013;86: 632–644. 10.1016/j.bcp.2013.06.021 23817078

[21] Huang GJ, Pan CH, Liu FC, Wu TS, Wu CH. Anti-inflammatory effects of ethanolic extract of *Antrodia salmonea* in the lipopolysaccharide-stimulated RAW246.7 macrophages and the k-carrageenan-induced paw edema model. Food Chem Toxicol. 2012;50: 1485–1493. 10.1016/j.fct.2012.01.041 22326970

[22] Castardo JC, Prudente AS, Ferreira J, Guimarães CL, Monache FD, Filho VC, et al Anti-inflammatory effects of hydroalcoholic extract and two biflavonoids from *Garcinia gardneriana* leaves in mouse paw oedema. J Ethnopharmacol. 2008;118: 405–411. 10.1016/j.jep.2008.05.002 18555627

[23] Molyva D, Kalokasidis K, Poulios C, Dedi H, Karkavelas G, Mirtsou V, et al Rupatadine effectively prevents the histamine-induced up regulation of histamine H1R and bradykinin B2R receptor gene expression in the rat paw. Pharmacol Rep. 2014; 66: 952–955. 10.1016/j.pharep.2014.06.008 25443720

[24] Barbosa AGR, Oliveira CDM, Lacerda-Neto LJ, Vidal CS, Saraiva RA, da Costa JG, et al Evaluation of chemical composition and antiedematogenic activity of the essential oil of *Hyptis martiusii* Benth. Saudi J Biol Sci. 2015;24: 355–361. 10.1016/j.sjbs.2015.10.004 28149173 PMC5272940

[25] Oliveira RB, Chagas-Paula DA, Secatto A, Gasparoto TH, Faccioli LH, Campanelli AP, et al Topical anti-inflammatory activity of yacon leaf extracts. Braz. J. Pharmacogn. 2013;23: 497–505.

[26] Leite GO, Leite LHI, Sampaio RS, Araruna MKA, Rodrigues FFG, de Menezes IRA, et al Modulation of topical inflammation and visceral nociception by *Vanillosmopsis arborea* essential oil in mice. Biomed Prev Nutr. 2011;1: 216–222.

[27] Ibrahim B, Sowemimo A, Rooyen A, Van de Venter M. Anti-inflammatory, analgesic and antioxidant activities of *Cyathula prostrata* (Linn.) Blume (Amaranthaceae). J. Ethnopharmacol. 2012;141: 282–289. 10.1016/j.jep.2012.02.032 22387161

[28] Karram EH, Harper GP. Collagen Degradation Within Subcutaneous Air Pouches *In Vivo*: The Effects of Proteinase Inhibitors. J Pharmacol Toxicol Methods. 1995;34: 97–102. 8563038 10.1016/1056-8719(95)00042-g

[29] Ozdol NC, Melli M. Formation of 8-isoprostaglandin F2α and prostaglandin E_2_ in carrageenan-induced air pouch model in rats. Eur J Pharmacol. 2004;506: 189–197. 10.1016/j.ejphar.2004.10.050 15588740

[30] Maleki-Dizaji N, Eteraf-Oskouei T, Fakhrjou A, Maljaie SH, Garjani A. The effects of 5HT_3_ receptor antagonist granisetron on inflammatory parameters and angiogenesis in the air-pouch model of inflammation. Int Immunopharmacol. 2010;10: 1010–1016. 10.1016/j.intimp.2010.05.013 20646986

[31] Marroquin-Segura R, Flores-Pimentel M, Carreón-Sánchez R, Garcia-Burciaga MM, Mora-Guevaraa JLA. The effect of the aqueous extract of *Helietta parvifolia* A. Gray (Rutaceae) stem bark on carrageenan-induced paw oedema and granuloma tissue formation in mice. J Ethnopharmacol. 2009;124: 639–641. 10.1016/j.jep.2009.06.004 19524657

[32] Zhang CX, Dai ZR, Cai QX. Anti-inflammatory and antinociceptive activities of *Sipunculus nudus* L. extract. J Ethnopharmacol. 2011;137: 1177–1182. 10.1016/j.jep.2011.07.039 21807085

[33] Xu Q, Wang Y, Guo S, Shen Z, Wang Y, Yang L. Anti-inflammatory and analgesic activity of aqueous extract of *Flos populi*. J Ethnopharmacol. 2014;152: 540–545. 10.1016/j.jep.2014.01.037 24508857

[34] Meshram GG, Kumar A, Rizvi W, Tripathi CD, Khan RA. Evaluation of the anti-inflammatory activity of the aqueous and ethanolic extracts of the leaves of *Albizzia lebbeck* in rats. J Tradit Complement Med. 2015; 2015: 1–4. 10.1016/j.jtcme.2014.11.038 PMC4833457 27114941

[35] Xu S, Hermanson DJ, Banerjee S, Ghebreselasie K, Clayton GM, Garavito RM, et al Oxicams Bind in a Novel Mode to the Cyclooxygenase Active Site via a Two-water-mediated H-bonding Network. J Biol Chem. 2014;289: 6799–6808. 10.1074/jbc.M113.517987 24425867 PMC3945341

[36] Berman HM, Westbrook J, Feng Z, Gilliland G, Bhat TN, Weissig H, et al The Protein Data Bank. Nucleic Acids Res. 2000;28: 235–242. Available from: http://www.rcsb.org. 10592235 10.1093/nar/28.1.235 PMC102472

[37] Morris GM, Huey R, Lindstrom W, Sanner MF, Belew RK, Goodsell DS, Olson AJ. Autodock4 and AutoDockTools4: automated docking with selective receptor flexiblity. J Comput Chem. 2009;16: 2785–2791. 10.1002/jcc.21256 PMC2760638 19399780

[38] ACD/ChemSketch, version 12.01, Advanced Chemistry Development, Inc., Toronto, ON, Canada, www.acdlabs.com, 2015. Available from: http://www.acdlabs.com.

[39] GaussView, Version 6, Dennington R, Keith TA, Millam JM. Semichem Inc., Shawnee Mission, KS, 2016.

[40] Gaussian 09, Revision A.03, Frisch M.J, Trucks GW, Schlegel HB, Scuseria GE, Robb M A, Cheeseman JR, et al. Gaussian, Inc., Wallingford CT, 2016.

[41] Pettersen EF, Goddard TD, Huang CC, Couch GS, Greenblatt DM, Meng EC, et al UCSF Chimera—a visualization system for exploratory research and analysis. J Comput Chem. 2004;25: 1605–12. 10.1002/jcc.20084 15264254

[42] Morris GM, Goodsell DS, Halliday RS, Huey R, Hart WE, Belew RK, et al Automated docking using a Lamarckian genetic algorithm and an empirical binding free energy function. J Comp Chem. 1998;19: 1639–1662.

[43] Bardin L. The complex role of serotonin and 5-HT receptors in chronic pain. Behav Pharmacol. 2011;22: 390–404. 10.1097/FBP.0b013e328349aae4 21808193

[44] Abbott FV, Hong Y, Blier P. Activation of 5-HT_2A_ receptors potentiates pain produced by inflammatory mediators. Neuropharmacology. 1996;35: 99–110. 8684602 10.1016/0028-3908(95)00136-0

[45] Brenchat A, Romero L, Garcia M, Pujol M, Burgueno J, Torrens A, et al 5-HT_7_ receptor activation inhibits mechanical hypersensitivity secondary to capsaicin sensitization in mice. Pain;141: 239–247. 10.1016/j.pain.2008.11.009 19118950

[46] Cury Y, Picolo G, Gutierrez VP, Ferreira SH. Pain and analgesia: The dual effect of nitric oxide in the nociceptive system. Nitric Oxide. 2011;25: 243–54. 10.1016/j.niox.2011.06.004 21723953

[47] Cunha TM, Roman-Campos D, Lotufo CM, Duarte HL, Souza GR, Verri-Junior WA, et al Morphine peripheral analgesia depends on activation of the PI3Kgamma/AKT/nNOS/NO/KATP signaling pathway. Proc Natl Acad Sci USA. 2010;107: 4442–4447. 10.1073/pnas.0914733107 20147620 PMC2840166

[48] Gold MS, Gebhart GF. Nociceptor sensitization in pain pathogenesis. Nat Med. 2010; 16: 1248–1257. 10.1038/nm.2235 20948530 PMC5022111

[49] Ricciotti E, FitzGerald GA. Prostaglandins and Inflammation. Arterioscler Thromb Vasc Biol. 2011;31: 986–1000. 10.1161/ATVBAHA.110.207449 21508345 PMC3081099

[50] Schreiber R, Zechner R. Lipolysis meets inflammation: arachidonic acid mobilization from fat. J Lipid Res. 2014;55: 2447–2449. 10.1194/jlr.C055673 25332433 PMC4242437

[51] Trott O, Olson AJ. Software news and update AutoDock Vina: improving the speed and accuracy of docking with a new scoring function, efficient optimization, and multithreading. J Comp Chem. 2010;31: 455–461. 19499576 10.1002/jcc.21334 PMC3041641

[52] Lipinski CA. Lead- and drug-like compounds: The rule-of-five revolution. Drug Discov Today Technol 2004;1: 337–341. 10.1016/j.ddtec.2004.11.007 24981612

